# Embracing leadership of local actors and community in disaster risk reduction of Yogyakarta

**DOI:** 10.4102/jamba.v16i1.1679

**Published:** 2024-09-16

**Authors:** Dina Ruslanjari, Retno A.P. Putri, Diana Puspitasari, Sigit Sulistiyo

**Affiliations:** 1 Master of Disaster Management, Graduate School, Universitas Gadjah Mada, Yogyakarta, Indonesia

**Keywords:** disaster preparedness villages (KSB), local leadership, disaster risk reduction, social capital, community resilience

## Abstract

The Disaster Preparedness Village (Kampung Siaga Bencana or KSB) in Special Region of Yogyakarta Province (DIY) is a community that aims to increase community preparedness and resilience to disasters through collaboration between the local government, non-government organisations and the community. This research examines the sustainability factors of KSB, namely the role of local leadership and government collaboration in disaster risk reduction (DRR) efforts. The method used in this research is a qualitative method and Strength, Weakness, Opportunity and Threat (SWOT) analysis, then using a diamond model to visualise the role of local leadership and the role of government. The results of this research show the importance of local leadership role in the sustainability of KSB. Factors contributing to the success of DIY KSB include independence, fulfillment of social granary logistics and effective two-way communication between KSB management and the village government (Kalurahan). The study supports that effective local leadership empowers the community, influences resource utilisation and facilitates collaborative efforts with the village government and volunteer organisations to address disaster management priorities. In addition, the most significant shortcomings in community leadership based on trust cultural value will negatively impact their sustainability and activeness if the local actors as mobilisers are inactive, and there is no regeneration of active new leaders. The Umbulharjo KSB case study exemplifies effective local leadership, mainly through Jagabaya, the chairman of KSB management. Strong local leadership is a fundamental element in developing a cohesive community actively involved in DRR efforts.

**Contribution:**

The study reveals that local leadership significantly contributes to the development and sustained activity of KSB. The research concludes by emphasising the multifaceted nature of local leadership, considering various perspectives and the community’s values and goals. The diamond model illustrates the intersection between societal opportunities and government support, demonstrating the realisation of goals through effective local leadership.

## Introduction

Disasters not only impact losses and casualties on society but also disrupt social life (Fang et al. 2018). The intensity of disaster impacts varies according to the conditions of the affected region and sector (Shaari, Karim & Hasan-Basri 2017). Disaster management efforts are carried out in a multisector manner and involve community participation through disaster communities. Community participation is necessary because the community is the subject and object of national development and disaster management (Mariana, Fikri & Syahrina 2020; Syafrizal 2013).

The disaster community is a forum that plays a strategic role in community resilience in carrying out the stages of the disaster cycle (Syahrizal 2019). Community-based disaster management encourages a disaster risk reduction (DRR) approach at the local level to change the disaster management paradigm from an emergency response to a disaster prevention and DRR paradigm in the community (Habibullah 2013). Considering Law number 24 of 2007 concerning Disaster Management, Government Regulations and Head Regulations, which have emphasised and included the concept of DRR.

Community-based disaster management policies are accommodated in the Regulation of the Minister of Social Affairs of the Republic of Indonesia Number 128 of 2011 concerning Disaster Preparedness Villages (KSB). Its establishment aims to protect the community from various disaster threats and risks by organising community-based disaster prevention and management activities through the use of natural and human resources in the local environment (Belanawane 2015). According to Habibullah (2013), the concept of KSB does not only refer to the definition of a village but also to a community-based disaster management forum or institution that can be based in sub-districts, villages, sub-districts and even hamlets. Cahyono (2019) explains in KSB that awareness regarding disasters and their impacts is a significant component for communities to adapt to difficult situations that require living with disasters based on experience and local wisdom.

According to the United Nations Office for Disaster Risk Reduction (2019), many countries face challenges in implementing DRR plans such as overly general DRR plans that make it difficult to guide concrete actions, as well as implementation constraints because of limited budgets, a lack of coordination among institutions and immature DRR guidelines. Several challenges in implementing DRR strategies at the local level include many aspects, namely early warning mechanisms, conducting disaster risk assessments, socialising institutional regulations related to disaster management, disaster education and reducing underlying risk factors (Fahmi & Hizbaron 2023). Kampung Siaga Bencana as disaster local community also faces similar challenges; however, each KSB in Indonesia has distinct issues based on local factors.

The role of KSB in Indonesia’s DRR efforts involves engaging communities in enhancing local resilience to disaster events. The availability of emergency disaster equipment and conducting simulations are commonly known and even practiced by the community (Dimaputri & Mujahidin 2023). Furthermore, Rumambi, Sari and Utami (2023) explain that some successes of the KSB programme in local DRR efforts include increasing public awareness, improving disaster-resistant infrastructure, enhancing community preparedness and reducing disaster losses.

The success of disaster management at the local level is greatly influenced by the strength of regional leaders. Social system support and efficient cultural mechanisms organise communities to increase local capacity in dealing with disasters (Harjono 2014). The existence of superior human resources such as local figures is very much needed in disaster management. The role of local leadership, especially for community leaders, is an important component in various DRR actions based on local wisdom. According to Harjono (2014), local leaders in dealing with disaster situations tend to be heard by the community rather than leaders from the national level, thereby emphasising the significance of regional-level leadership in the context of DRR practices is considered more effective in carrying out community-based activities and also promoting values, such as the value of local wisdom.

Based on data from the DIY Social Agency in 2023, there are 301 out of 393 sub-districts and 45 sub-districts included in disaster-prone areas. The DIY, which has multiple disaster risks, should be able to optimise the leadership role of local figures through KSB that have been formed in various DRR actions. Aspects of local wisdom that are still strong in various regions have great potential for achieving community resilience in facing disasters. This research aims to analyse the context of local leadership and identify strategies for success in achieving a Disaster Resilient Community in implementing the KSB programme.

## Research methods and design

### Study area

**FIGURE 1:**
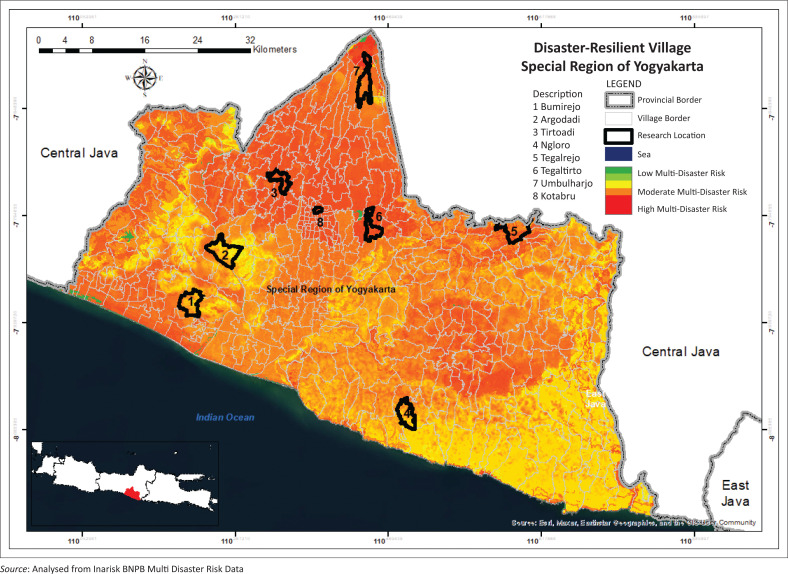
Distribution of the KSB (study areas) on disaster risk map in Yogyakarta.

The research sites employed a purposive sampling technique, taking into account the selection of the KSB in DIY (Given 2008). The number of KSBs that have been formed in DIY has 65 KSBs, which are divided into four categories, namely not developing, moderately developing, well developing and very well developing. The selection of KSB samples as research objects was based on the representation of each category and type of disaster in DIY. The research locations cover eight village-level areas (Kalurahan) in five districts or cities in the DIY (Figure 1).

### Data collection

This research uses qualitative case study analysis, which focuses on the relationship between local leadership and successful efforts in the KSB programme as the research object. A single case study qualitative method was used to explore more deeply the factors and indicators in local leader interactions (Lin, Kelemen & Kiyomiya et al. 2016). The qualitative case study research strategy focuses on analysing phenomena in a broader context and comes from various data sources (Yin 2014).

Primary data collection in this research was carried out by direct observation of KSB facilities, member interactions and their work areas, in-depth interviews, focus group discussion (FGD) and analysis of documents related to KSB including social service reports, previous research and regulations related to KSB. According to Lincoln and Guba (1985) in Salim and Syahrum (2012), data collection using interviews, observation and document study can support and complement each other in fulfilling the data required in the research focus. Creswell et al. (2017) state that there are four procedures for collecting data, that is qualitative observation, qualitative interviews and study of documents, both public documents and private documents, during the research process. Data collection was carried out for 4 months.

A total of 28 KSB members participated for two focus groups and two participants for in-depth interview were included in this study (Table 1). The participants who met criteria such as: (1) have direct knowledge about KSB, (2) had experience in KSB and (3) were capable of recalling various events during KSB activities in DIY were used for sampling. In-depth interviews are conducted to explore the behaviours, beliefs and experiences of respondents or key informants regarding a KSB (Bastian, Winardi & Fatmawati 2018). The FGD process is used to build a scientific foundation with the community in aspects of leadership and local wisdom (Zulfadrim, Toyoda & Kanegae 2018). The key informants in this research include Social Agency officials in DIY and stakeholders from the sample villages. Field observations were carried out to observe the existing conditions of the KSB Umbulharjo, both in terms of activeness and local leadership in implementing DRR. Researchers conducted systematic observations and documentation of KSB activities throughout the village to identify and provide empirical evidence of the actions and interactions within KSB organisations operating in highly disaster-prone areas, where KSB Umbulharjo is an area exposed to the danger of Merapi Volcano on a frequent basis. Table 1 provides the general information of the participants for the Key Informant interviews and FGDs.

**TABLE 1:** General information of the participants.

Participants code	Villages	Positions	Gender
**In-depth interview**			
R1	-	Head of KSB at social agency officials in province level	Male
R2	-	Social agency officials	Male
**Focus Groups 1**			
R3	Bumirejo	Head of KSB	Male
R4	Bumirejo	KSB staff	Male
R5	Argodadi	Head of KSB	Male
R6	Argodadi	KSB staff	Male
R7	Ngloro	Head of KSB	Male
R8	Ngloro	KSB staff	Male
R9	Tirtoadi	Head of KSB	Male
R10	Tirtoadi	KSB staff	Male
R11	Tegaltirto	Head of KSB	Male
R12	Tegaltirto	KSB staff	Male
R13	Umbulharjo	Head of KSB	Male
R14	Umbulharjo	KSB staff	Female
R15	Tegalrejo	Head of KSB	Male
R16	Tegalrejo	KSB staff	Male
R17	Kotabaru	Head of KSB	Male
R18	Kotabaru	KSB staff	Male
**Focus Groups 2**			
R19	Umbulharjo	Head of KSB	Male
R20	Umbulharjo	KSB member	Male
R21	Umbulharjo	KSB member	Male
R22	Umbulharjo	KSB member	Female
R23	Umbulharjo	KSB member	Female
R24	Umbulharjo	KSB member	Female
R25	Umbulharjo	KSB member	Female
R26	Umbulharjo	KSB member	Female
R27	Umbulharjo	KSB member	Female
R28	Umbulharjo	KSB member	Female
R29	Umbulharjo	KSB member	Female
R30	Umbulharjo	KSB member	Female

The secondary data collected regarding KSB in DIY, related to local leadership in disaster management that has been implemented in the past or is currently being implemented, is enriched through literature studies as references. These literature studies include previous research, news articles, or government report documents. Secondary data collection and relevant information for use in this research include identifying leadership models and local wisdom that are appropriate to the existing conditions (Hutagalung & Indrajat 2020).

### Data analysis

Data analysis and data collection were conducted simultaneously. All recorded voices and detailed notes were transcribed and analysed via content analysis. The research employs two types of data analysis, leadership analysis and SWOT analysis. Leadership analysis is conducted to recognise and define leadership in the implementation of KSB. Based on Fairholm’s (1998) conception of leadership, there are five perspectives. This perspective defines leadership from a personal perspective that wants to be seen internally and externally as well as the form of success resulting from this leadership.

The definition of leadership is:

- Leadership in the form of (scientific) scientific management
- Leadership in the form of management excellence
- Leadership in the form of value displacement activity
- Leadership in the form of a culture of trust, and
- Leadership in the form of spiritual values (whole soul).

The cultural conditions in DIY that are strong in Javanese culture and values seen from the five leadership perspectives are suitable for analysis with a leadership perspective in the form of a culture of trust. In the leadership analysis, some elements must be fulfilled to be included in the leadership perspective used in the KSB.

Meanwhile, SWOT analysis is utilised to scrutinise the strategic plans and management techniques employed by KSB in mitigating disaster risks at the local level. After data extraction from all focus group and interview texts, the information was condensed into a single text. Keywords, statements or phrases that exemplified what local leadership at the KSB level expected were observed and highlighted. To generate categories for leadership and SWOT analysis, common ideas in the text were sorted and classified according to their differences and similarities. Crossing out repeated or similar words or phrases within the categories helped to eliminate redundancy in the number of words and phrases. Finally, after a few changes, the themes showed through. Participating in this process, all authors talked about how the SWOT and leadership analysis were developed.

#### Leadership in disaster management

Local leadership is analysed from a technical and soft skills perspective as a form of active leadership. Technical capabilities are used to identify programmes that can run objectively and are relevant to the government. Soft skills demonstrate leadership that can manage efficiently according to the socio-cultural context involving stakeholders (Lin et al. 2017).

The analysis of local leadership in this research aims to identify findings related to leadership style in the field. The role of local leadership is crucial in the disaster management cycle. Based on research by Leadbeater (2013), there are six focus areas used to identify leadership practices in the community: time, leadership, relationships, capacity, local knowledge, and communication.

#### Strength, weakness, opportunity and threat analysis for strategic plan of the local community

Strength, weakness, opportunity and threat analysis is a systematic analytical approach utilised to identify and evaluate the factors of ‘strengths’, ‘weaknesses’, ‘opportunities’ and ‘threats’. This method is commonly applied in formulating strategies for companies, organisations, institutions or communities (Anjasni 2013; Mukhlasin & Pasaribu 2020). The primary goal is to conduct a thorough analysis and examination of the components involved in the KSB strategy to ensure success. The choice of SWOT analysis is justified by its effectiveness and logical analytical capabilities Sabbaghi & Vaidyanathan (2004) and as explained by Chang, H.H. & Huang, W.C. (2006). It serves as an approach to identify both internal and external factors in the research object’s process or phenomenon (Leiber, Stensaker & Harvey 2018). The four factors, specifically ‘strengths’, ‘weaknesses’, ‘opportunities’ and ‘threats’, are instrumental in analysing the implementation of various KSB programmes:

- Strength is used to determine the internal assets that help in meeting demands and facing threats such as motivation, technology and the like.
- Weakness shows internal factors that hinder us in facing threats and meeting demands.
- Opportunity is used to describe the conditions or circumstances of external factors that can support meeting demands.
- Threat is defined as an external condition or circumstance that threatens or is disadvantageous in fulfilling demands.

After analysing the KSB sustainability factors using SWOT, the relationship between the role of local leadership and government collaboration through the village government can be modelled using Porter’s Diamond Model. The diamond model can be used to determine the competitive advantage of a nation or community group and to assess whether a country or region has the potential to serve as a foundation for growth in a specific sector (Porter 1990).

### Ethical considerations

This article followed all ethical standards for research without direct contact with human or animal subjects.

## Results

### Leadership approaches for Kampung Siaga Bencana implementation

Based on the results, the Umbulharjo KSB implemented leadership through a culture of trust. Trust plays a pivotal role in organisational leadership, as followers willingly opt to align themselves with leaders, rather than being compelled to do so. The confidence bestowed by followers empowers leaders to exert influence. It serves as the cohesive force binding together the organisation, its initiatives and its personnel. Undoubtedly, interpersonal trust is indispensable for the functioning of any organisation. Moreover, leaders must acknowledge the profound significance of trust as they navigate the cultivation and administration of their organisation’s culture and persuade stakeholders to act in alignment with organisational objectives. This is demonstrated by the fulfillment of the trust culture leadership elements as evidenced by the views of respondents as follows:

‘Feel safe and residents feel he can be relied on and impartial when faced with a problem.’ (Respondent 22, KSB member, Female)

According to Fairholm (2002), in the trust culture leadership, there are elements used to define existing leadership, including:

- Ensure a culture conducive to mutual trust and integrated collective action
- Prioritisation of shared cultural values and values created through the culture of the behaviour of the group/organisation
- Form and maintain culture through a shared vision
- Sharing group/organisation governance
- Give appreciation to the group’s achievements
- Trust
- Team building
- Foster a shared culture.

An inherent aspect of cultivating a values-driven leadership environment is the notion that leaders are responsible for fostering a culture rooted in shared values, facilitating an environment where mutual trust enables collaboration. Leadership, therefore, encompasses both individual and group endeavours. A comprehensive grasp of leadership dynamics is attainable solely within cultures marked by common values and objectives. It is within harmonious cultural contexts that leaders can exert an influence on their followers.

### Strategic plan of the local community in disaster risk reduction

The strategies obtained from the SWOT analysis are presented in a table divided into four S-O strategies, four W-O strategies, four S-T strategies and four S-T strategies. The four strategies are structured based on the concentration of internal aspects in the form of strengths and weaknesses as well as external aspects of opportunities and challenges (Oktari et al. 2023). Strength, weakness, opportunity and threat analysis results can be seen in Table 2.

**TABLE 2:** Strength, weakness, opportunity and threat analysis of disaster preparedness village strategy in disaster risk reduction.

SWOT analysis	Strengths (S)	Weakness (W)
	S1. There are local figures, namely Jagabaya and Pinunjul who are role models for volunteers and the community S2. Trust in local wisdom S3. Involvement of sub-district heads or policy stakeholders S4. Knowledge about disaster	W1. Location access is difficult to reach W2. Individual characteristics of society W3. The budget has not been realised W4. Administrative obstacles

**Opportunities (O)**	**Strategy (S-O)**	**Strategy (W-O)**

O1. High sense of volunteerism O2. Potential participation of the younger generation in KSB O3. Existence of standard operating procedures (SOP) O4. Utilisation of social barns for activities with the community	SO1. Optimising the role of local figures in developing volunteer programmes SO2. Develop programmes that combine local wisdom with innovative ideas from the younger generation SO3. Integrating the involvement of sub-district heads or policy stakeholders to optimise the efficiency of SOP SO4. Increasing knowledge about disasters and training to increase capacity	WO1. Providing special training to volunteers to face geographical challenges in difficult areas WO2. Using social barns as a platform to build social capital to encourage active participation from the community WO3. Involve sub-district heads/policy stakeholders to negotiate with KSB administrators regarding budget use WO4. Utilise existing SOPs to improve administrative processes to be more efficient

**Threats (T)**	**Strategy (S-T)**	**Strategy (W-T)**

T1. There is competition between volunteer communities T2. The volunteers who join KSB are also village officials T3. Coordination between the KSB management and sub-district administrators is still one-way T4. The application of local wisdom values is very thin	ST1. Using local wisdom and the influence of local figures to collaborate between volunteer communities ST2. Implementing involvement of sub-district heads/policy stakeholders by ensuring that the roles of volunteers in KSB and village officials are separate and clear. ST3. Utilise disaster knowledge to increase shared understanding of the importance of effective coordination and teamwork and build a transparent and communicative framework ST4. Increasing trust in local wisdom by integrating these values into KSB programmes	WT1. Develop special transportation systems or efficient route planning WT2. Hold training sessions and workshops to increase understanding of the importance of working as a team and encourage active participation from volunteers who are also village officials WT3. Develop a more efficient administrative system by strengthening financial supervision and reporting WT4. Increase public awareness of local wisdom through educational programmes, community meetings and cultural activities that encourage a deeper understanding of cultural heritage and traditional values

SWOT, strength, weakness, opportunity, and threat.

The results of field analysis and identification show that KSB’s success in sustainability and activeness of DRR activities is optimising local leadership in developing volunteer programmes (SO1, ST1). Implementation of these two main strategies in disaster management at the community level to effectively mobilise the community down to the family level living in disaster-prone areas such as the KSB DIY Province area. This can be seen from the participation of KSB members and volunteers in the field who can spread awareness of hazards in the surrounding environment.

There are obstacles found in realising the Strategic Plan of The Local Community in DRR related to the importance of collaboration between KSB and the government. These obstacles include funding support that has not been realised and volunteer administration problems that have not been distributed evenly to all registered volunteers in the community. As said by respondent 11 in the focus group discussion:

‘KSB has become less active because it has never received funding from the sub-district. The only support received was during the formation of the KSB by the Social Service.’ (Respondent 9, Head of KSB, Male)

These problems were also encountered in several KSBs in DIY. The root of the problem is that there is no uniform monitoring and evaluation of KSB in DIY so government support cannot be realised.

Local wisdom such as jimpitan, merti desa, gotong royong and local beliefs regarding sacred trees are also the main focus of KSB’s success strategy, namely developing programmes that combine local wisdom with innovative ideas from the younger generation (SO2, ST4). Some ways to implement this strategy are the introduction of local wisdom, intergenerational collaboration, the development of adaptive technology and the use of social media and education (ST3). Apart from that the factors that support the success of the DIY Disaster Preparedness Village (KSB) are the independence of the KSB, fulfillment of social granary logistics and two-way communication between the KSB management and the village government (Kalurahan).

The local leadership implemented by KSB in DIY is based on a culture of trust, a case study used in this research, namely the Umbulharjo KSB Village case study. Judging from KSB opportunities, namely the trust of local figures who are role models for volunteers and the community and local wisdom, it is known that leadership with a trust culture perspective can contribute to the development of disaster alert villages.

Social capital ‘Gotong royong’ in the form of strong relationships within the village community makes it easier to mobilise the community and share information during crisis and post-disaster conditions (WO2). The family nature is an effective mechanism for sharing information informally among KSB volunteers. This can be seen in the form of strategies based on local wisdom in DIY such as cooperation and ‘merti desa’. This entire process is an asset resulting from routine face-to-face interaction in village communities, a high level of trust and having the same values in togetherness (Casprini et al. 2017; Oktari et al. 2021, 2023).

### Trust cultural leadership of the local community

This study shows that local leadership has a fundamental role in KSB in achieving community resilience as an effort to reduce disaster risk. Strong local leadership will build a solid community and feel more directly involved in DRR efforts, especially at the village level:

‘A communication and information network formed to coordinate with village officials which is very solid, making it easier for local communities to receive disaster management quickly and appropriately.’ (Respondent 19, Head of KSB, Male)

According to Saha (2021), local leadership includes a person’s ability to influence and empower the resources of their community, so that they can translate every vision into reality by optimising efforts to achieve goals.

Good local leadership in KSB can mobilise the community by setting handling priorities and empowering the community. Furthermore, the role of local leaders who are trusted together with the village government and volunteer organisations can work together to find and implement solutions that support priorities in disaster management according to actual conditions. One of the important roles of leaders, especially in an emotional and stressful environment as a sign of a crisis, is to be able to provide psychological security so that community members and disaster management teams can openly discuss their ideas, questions and concerns without feeling afraid of the consequences that might arise. This of course helps the team network in understanding the situation and how to handle it through a healthy discussion process (D’Auria & De Smet 2020).

The diamond model can specifically describe the results of the SWOT analysis of what factors can influence KSB’s sustainability. It can explain how the role of local leadership and village government collaboration are related.

Umbulharjo KSB is an example of KSB for observation. The local figure who is considered a leader in KSB Umbulharjo is Jagabaya who is also the chairman of the KSB management. According to the community, he is a wise and very nurturing figure and always provides solutions in solving problems in the village. Meanwhile, according to KSB members and volunteers, he is an example through what he did; therefore the previous KSB members or volunteers became interested in taking part in KSB activities and even becoming administrators:

‘Pinunjul, Jagabaya, or local figures who are trusted by the community have enough influence on the community to care more about the importance of disaster science and participate actively in the disaster community, namely KSB.’ (Respondent 11, Head of KSB, Male)

Trust cultural leadership of the local community could be referring to the significance of trustworthy leadership that is culturally sensitive and responsive to the needs and values of a specific local community. Elliott (2019) said that the efficacy of empowerment relies on the level of authority that communities possess in making decisions regarding matters that impact them, and this authority is delegated, in this instance, by local authorities.

The KSB that is categorised as very developed, well developed and quite developed shows that the role of local leadership in DRR actions is that it can support the development of the KSB to remain active, proven at least by the existence of socialisation activities regarding the existence of the KSB.

Local leadership can be viewed from various points of view and depends on the situation, values and goals of the community. The figures of concern are Pinunjul who has authority as a local leader, Dukuh who plays an active role in the management of the KSB and Jagabaya who can be an intermediary in conveying the needs of the KSB with the sub-district. The coordination carried out is by distributing information or direction according to the KSB organisational structure and communication network. According to Porter (1990) in the OECD Regional Development Papers, the local leadership can play a catalytic role in community development with, in some cases, active collaboration between local stakeholders (Kalurahan).

**FIGURE 2:**
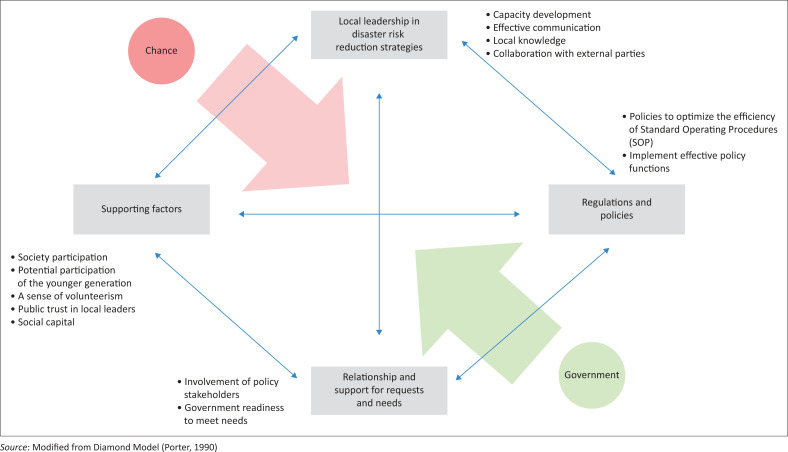
Diamond model in implementing local leadership.

Considering the diamond model in implementing local leadership (Figure 2), there is a meeting point between the opportunities that exist in society and the role of support that can be provided by the government in reducing disaster risk. In this model, it can be also seen that the goals to be achieved can be realised with local leadership; based on a study by Wang et al. (2014), sustainability at a local level encompasses more than just environmental initiatives such as energy conservation. It also includes policy endeavours to engage communities, enhance organisational capacity and promote widespread adoption.

The meeting point in the diamond model is the role of the local leadership (chance) and the role of government (government); if implemented by optimising each function, an effective and efficient DRR strategy will be formed. The role of local leadership in the diamond model is to provide encouragement to the community to participate in DRR and mediate the aspirations conveyed by the community to the government. Meanwhile, the government’s role is to provide support in accordance with community needs and regulations. So, with this collaboration, inclusiveness will be achieved in the disaster management sector.

## Conclusion

The application of local leadership in DRR strategies can be developed by analysing the strengths, weaknesses, opportunities and challenges at the KSB community level. This is carried out to find out which KSB management has been successfully adopted and developed by other KSBs according to their respective conditions. This study can produce four advantages, including the presence of local figures who serve as role models for volunteers and the community, trust in local wisdom, the involvement of policy stakeholders and the existence of disaster knowledge. As for the four disadvantages, they encompass difficult access to locations, heterogeneous characteristics of community individuals, limited disaster management budgets and administrative obstacles.

Four opportunities successfully identified include the high spirit of volunteerism, the potential participation of the younger generation in KSB, the existence of standard operating procedures and the utilisation of social barns for community activities. Meanwhile, the four challenges in implementing KSB in DIY include competition between volunteer communities, volunteers joining KSB also holding village government positions, one-way coordination between KSB management and administrators and the minimal application of local wisdom values within the community.

Model for implementing local leadership reveals a convergence point between societal opportunities and government support in reducing disaster risk. It emphasises the accomplishment of goals through effective local leadership, that sustainability at a local level comprises not only more than just environmental efforts but also policy involvement, organisational capacity building and widespread adoption promotion within communities. The success of KSB illustrates the critical role of local leadership, demonstrating its multidimensional impact on community development, active collaboration and achieving larger sustainability goals. Future research can be conducted to investigate the existing strategic priorities to determine their effectiveness in society and the impact of the sustainability of these leadership values. It can be concluded that the existing KSB in DIY are strong and effective in disaster management and management activities, but there is still a need for improving strategies to overcome existing weaknesses and threats. The results of this analysis are expected to serve as a reference for organisational management and sustainability in formulating appropriate strategies in dealing with the existing obstacles and threats on leadership context.
